# Nutritional Assessment of Hospital Meals by Food-Recording Applications

**DOI:** 10.3390/nu14183754

**Published:** 2022-09-11

**Authors:** Katsumi Iizuka, Takuma Ishihara, Mayuka Watanabe, Akemi Ito, Masayoshi Sarai, Ryoji Miyahara, Atsushi Suzuki, Eiichi Saitoh, Hitomi Sasaki

**Affiliations:** 1 Department of Clinical Nutrition, Fujita Health University, Toyoake 470-1192, Japan; 2 Innovative and Clinical Research Promotion Center, Gifu University Hospital, Gifu 501-1194, Japan; 3 Food and Nutrition Service Department, Fujita Health University Hospital, Toyoake 470-1192, Japan; 4 International Medical Center, Fujita Health University Hospital, Toyoake 470-1192, Japan; 5 Department of Endocrinology, Diabetes and Metabolism, Fujita Health University, Toyoake 470-1192, Japan; 6 Department of Rehabilitation Medicine I, School of Medicine, Fujita Health University, 1-98 Dengakubo, Kutsukake-cho, Toyoake 470-1192, Japan

**Keywords:** mobile food records, hospital food

## Abstract

Mobile food records are currently used to determine the nutrition of healthy subjects. To determine the accuracy of such records, we evaluated the nutritional composition of a test meal (noodles and fruit juice) and a hospital meal (Japanese set meal) using two types of mobile food records. Eighteen healthy subjects (2 males and 16 females) were enrolled. Using these diets and validated nutrient-composition information, we evaluated the accuracy of the dietary assessments made by two dietary-record applications, Asken^®^ and Calomeal^®^, over 5 days. For the test meal, the values provided by the two applications were close to the actual values. In contrast, for the hospital meal, the values provided by the two applications were approximately 1.5 times higher than the actual values. A linear-mixed-model analysis showed that the total energy, carbohydrate, and salt contents were significantly overestimated in the hospital meal. Protein also tended to be overestimated, while the fat content was not significantly overestimated. Furthermore, the total energy and fat contents increased significantly over time. No association with age was observed. A comparison of the coefficients of variation (CVs) for each nutrient in the hospital meal indicated that the fat levels were significantly higher than those in the test meal. In conclusion, the accuracy of mobile food records depends on the type of meal. Our data will provide lessons for the use of meal-recording applications in special cases, such as hospital food.

## 1. Introduction

Currently, several diet-assessment methods are widely used: weighed food records, the 7-day record method, the recall method, and food frequency questionnaires (FFQs). However, these methods have some problems, including the time requirements for the subject and the proficiency of the questioner [1].

In recent years, mobile food-recording applications (apps) have been developed and are being used for health guidance and other purposes [2]. One advantage of mobile applications is that they are simple and less burdensome for users because determinations can sometimes be made by simply taking a picture or entering items. However, their accuracy as programs remains unclear.

In daily life, a number of unhealthy foods sold in convenience stores are widely distributed [3,4]. Many of these foods are packaged with information about the amounts of energy and nutrients. However, these foods are rich in sugars, fats, and NaCl, and they can sometimes increase the risk of obesity-related diseases [5]. In contrast, hospital food has an appropriate energy level, and the carbohydrate:protein:fat ratio is calculated by a nutritionist [6,7]. In particular, decreasing the NaCl and fat contents is beneficial for the treatment of hypertension, cardiovascular disease, and diabetes mellitus [5]. Therefore, hospital food is a special diet with limited salt and fat contents in Japan.

In this study, our objective was to determine whether the food-recording-app errors differed for different types of meals. At the same time, we also examined the interindividual and diurnal variations. As no study has ever evaluated the nutritional value of hospital food using a meal-recording app, this study will provide lessons for the use of meal-recording apps in special cases, such as hospital food.

## 2. Materials and Methods

### 2.1. Mobile Food-Recording Application

**Figure 1:**
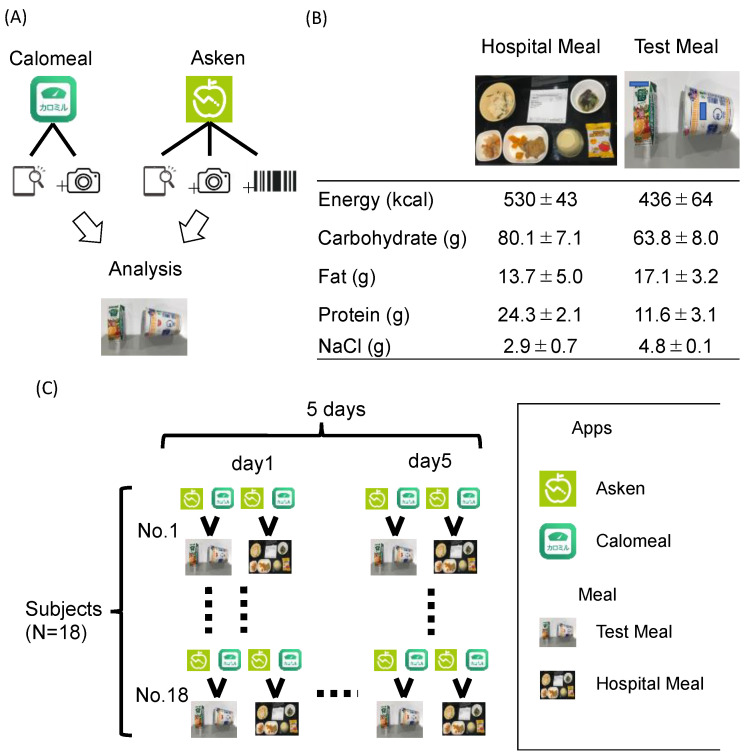
The characteristics of the Calomeal and Asken apps and the procedure of this study. (**A**) The Calomeal and Asken apps have image-analysis functions based on photos and food-search functions based on names. In addition, the Asken app has a search function based on product barcodes. Subjects were allowed to use all photo analyses, searches by product name, and barcode searches (Asken only) for analysis. In this study, all functions were used to analyze meals rather than specific functions. For example, if the photo analysis showed the wrong food, then it could be corrected by manually entering the name or by scanning the barcode. (**B**) Comparison between the hospital meal and test meal. Data represent the mean ± S.D. (n = 5). (**C**) Procedure of this study. Subjects were analyzed using two meal-recording apps against a test meal. The hospital meal was served for lunch (different contents each day, but approximately the same energy, protein, fat, carbohydrate, and salt contents), and the energy, protein, fat, carbohydrate, and salt contents were recorded and collected. This procedure was followed for 5 of the 14 days and analyzed; 25 people were recruited as the subjects, but 7 who did not have smartphones were excluded, and so the experiment was conducted with 18 people. Elderly subjects were not included in the study; those aged 20–64 years old were included. Note that the subjects did not eat the test meal; only the analysis was performed.

We evaluated the nutrient contents of these meals with two food-recording apps, Asken^®^ (Asken Corporation, Tokyo, Japan) and Calomeal^®^ (Life Log Technology, Inc., Tokyo, Japan), over 5 days (from 31 January to 10 February 2022). The reasons that we used these apps are that, in Japan, the Asken app is used by more than 5 million people, and the Calomeal app is used by more than 1.4 million people. The two food-recording apps include analysis by taking a photo and performing a search function according to the food name (Figure 1A), as these apps were designed with the assumption that both functions would be used.

Therefore, subjects were allowed to use all photo analyses, searches by product name, and barcode searches (Asken only) for analysis.

### 2.2. Foods

Two types of meals were used: a test meal and a hospital meal (1600 kcal per day). The test meal was a combination of ramen cups and vegetable juice, and the hospital meal (for lunch) was a Japanese set meal (main meal, main dish, side dish, and soup), with an energy content of approximately 550 kcal per meal (Figure 1B). The comparisons between the test food and the hospital food are shown in Figure 1B. Characteristically, the hospital food was lower in salt and fat and higher in protein and carbohydrates than the test food.

### 2.3. Subjects

Participants were recruited from Fujita Medical University staff (excluding dietitians) who were interested in the study; of the 25 applicants (M:7, F:18), 7 did not meet the inclusion criteria because they did not have smartphones. Therefore, 18 people participated in this study. The subjects were healthy 20–65-year-old individuals (41.4 ± 11.2 years old), including 16 women and 2 men. The total energy, carbohydrate, fat, protein, and salt contents were assessed using the two apps for two different meals for 5 days, and the information was recorded on a recording sheet (Figure 1C). Elderly individuals might not have been proficient in using the program. Therefore, we examined the number of times that the apps were used (times) and the subject age (>45 years old vs. <45 years old), in addition to the type of diet, to determine whether age affected the assessment of the energy intake and nutritional intake. Written consent was obtained from the subjects. The study was approved by the Ethics Committee of Fujita Medical College.

### 2.4. Statistics

The nutritional composition of the subjects at each meal over 5 days is presented as the mean ± standard deviation (SD) (Figure 1B). A linear mixed model was used to evaluate the effects of the meal type, time, and subject age (<45 years old, >45 years old) on the nutritional composition. A subject ID was included as a random effect in the model because repeated measurements were collected from each subject. The model included the participant ID as a random effect. Finally, the coefficient of variation (CV) for each parameter was compared using the Mann‒Whitney U test. A two-tailed *p* < 0.05 was considered to be statistically significant. All statistical analyses were conducted using R software (version 4.1.1 Patched:R Foundation for Statistical Computing, Vienna, Austria; www.r-project.org) (accessed on 10 August 2021).

## 3. Results

**Figure 2:**
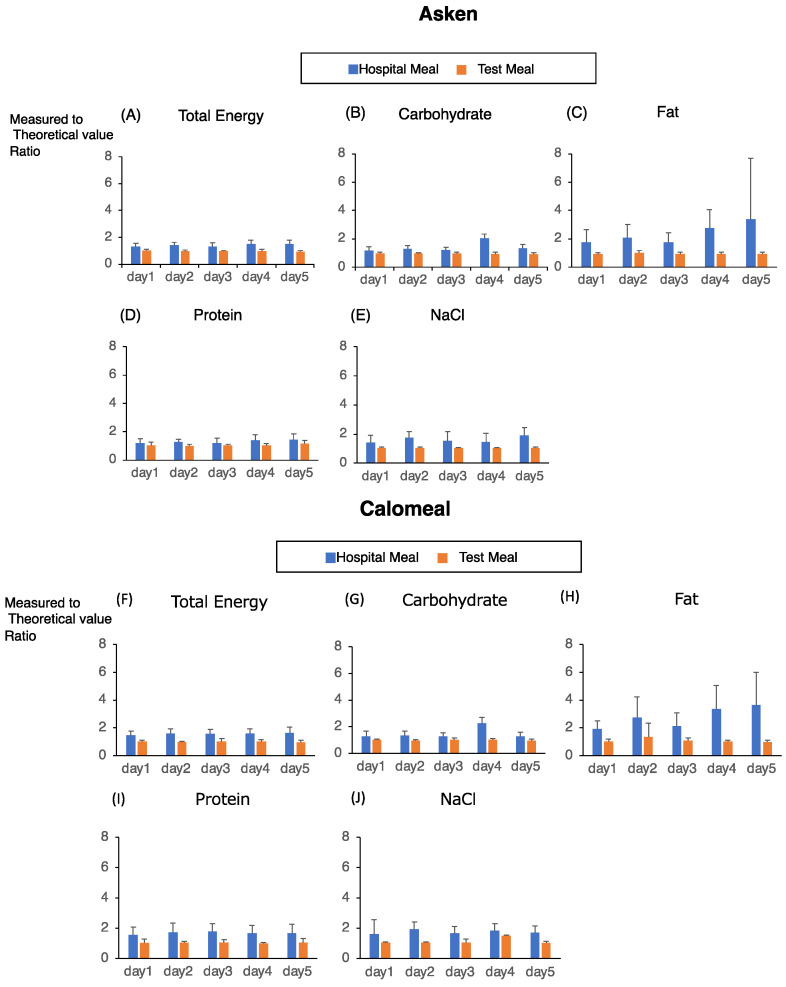
Measured vs. theoretical values of total energy and each nutrient for the hospital meal and test meal. The y-axis indicates the value obtained by dividing the actual value (measured value) obtained by the application by the true value (theoretical value) displayed on the food. If this value exceeded 1, then it indicated overestimation, and if it was less than 1, then it indicated underestimation. The blue lines show the hospital food, and the orange lines show the test food. Asken results are shown in the upper panel, and the unmatched results are shown in the lower panel. Each data point was measured by the Asken (**A**–**E**) or Calomeal (**F**–**J**) app. (**A**,**F**) total energy; (**B**,**G**) carbohydrates; (**C**,**H**) fat; (**D**,**I**) protein; (**E**,**J**) NaCl.

In this study, we used two types of meals with known energy and macronutrient values. First, we compared the measured and original values using the two apps. For the test meals (a cup of ramen and vegetable juice), the energy and nutrient contents could be measured almost exactly with both apps (Figure 2).

**Figure 3:**
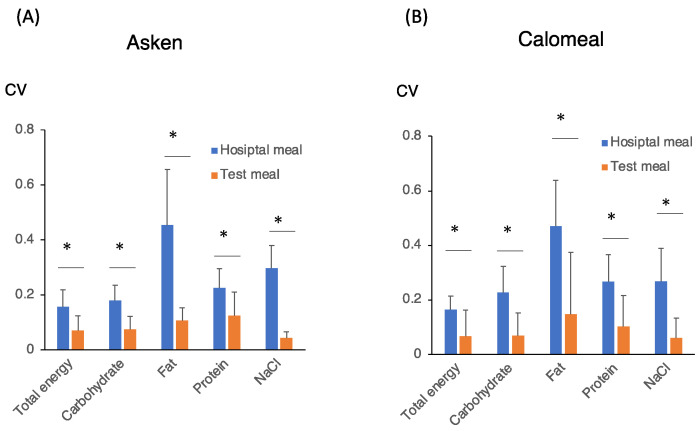
CV for total energy and each nutrient of the hospital meal and the test meal for 5 days. Data were measured by the Asken (**A**) and Calomeal (**B**) apps. The y-axis indicates the CV of the measured to theoretical value ratio. Data are represented as the mean ± SD (n = 18). * *p* < 0.05.

In contrast, for the hospital meals, the values measured by the two apps were approximately 1.5 times higher than the actual values of the diet. This finding was especially true for the fat and salt (Figure 2). The CV percentages for the total energy and each nutrient in the hospital meal were significantly higher than those in the test meal (Figure 3). In particular, the 5-day CV for fat in the hospital meal was the highest (at approximately 0.5) when measured by both methods. These results suggested that, regardless of the mobile food-recording app used, the subject-derived error of the values for the total energy and each nutrient was larger for the hospital diet and smaller for the test diet.

Next, we evaluated the different types of meals (meal) and the number of times that the apps were used (times) using a linear mixed model. Then, we examined the number of times that the apps were used (times) and the subject age (> 45 years old vs. < 45 years old), in addition to the type of diet, to determine whether age affected the assessment of the energy intake and nutritional intake. The linear mixed model showed that the types of food significantly affected the total energy, carbohydrate, and salt measurements (Table 1).

However, the type of food did not significantly affect the measurement of fat because of the higher CV (Table 1). In contrast, time significantly affected the measurement of energy and fats because the energy and fat contents in the hospital diets tended to be overestimated as the number of days after the start of the measurements increased (Figure 2 and Figure 3, Table 1). An interaction between time and the type of diet was found for the energy, carbohydrate, fat, and salt contents with the Asken app, and for the fat content with the Calomeal app (Table 1). No association with age was observed (Table 1). These results suggested that the measurement of the energy and each nutrient value, and especially fat, was dependent on both the type of meal and time. Thus, the type of food significantly affected the estimation of the total energy, carbohydrate, and salt intakes.

## 4. Discussion

In this study, the dietary records of the test meals in the two different mobile food record apps were very accurately measured. Conversely, the total energy and nutrients of the hospital meal might have been overestimated because hospital meals are designed to limit the total energy, and especially fats. These results suggested that the fat content is more difficult to measure than the carbohydrate and protein contents. Because hospital meals are low in salt and fat, we found that there is the possibility of overestimation when assessing hospital meals on a typical meal-recording application.

The most frequently used dietary-assessment approaches include weighed food records (in a community setting or in a closely monitored nutrition laboratory), estimated dietary records or food diaries, a single 24 h dietary recall, multiple 24 h dietary recalls, food frequency questionnaires (FFQs), and biomarkers, such as urinary nitrogen, the serum nutrient levels, or double-labeled water [1,2,3]. One disadvantage of food dietary records is that subjects tend to record less diligently with each additional day [1,2,3]. The disadvantage of a 24 h dietary recall is that it requires a skilled interviewer and sufficient recall ability. FFQs require a certain degree of literacy, rely on long-term recall ability, and are generally not as effective for determining the absolute intake of nutrients [1,2,3]. In this study, our reference meals included food with already known amounts of energy and nutrients. Therefore, in the validation of mobile food records, this method is more suitable than other methods [1,2,3].

Some papers have reported the validation of mobile record apps as a reference for other methods. Some have reported validation studies performed using mobile food records. In these cases, the references were 2-day 24 h dietary recalls (24HRs), FFQs, and a combination of the two [2,8,9,10]. The authors concluded that the dietary record apps underestimated the food consumption compared with traditional dietary-assessment methods. In some studies, the energy and nutrient intakes estimated by apps were compared with those calculated using the Standard Tables of Food Composition in Japan based on paper-based dietary recalls (DRs) (reference method) [9]. Shinozaki N. et al. concluded that the intakes of many nutrients were overestimated by the Asken and Calomeal apps and underestimated by the MyFitnessPal app. Although the reference methods were not the same and our methods using foods with already known nutrient values were more accurate than the methods used in other references, our data were compatible with the latter study. These results suggested that the differences between the reference data and measured values were dependent on the type of app and reference methods used. As with reference data, diet-recall methods could also overestimate the intake of total energy and each nutrient in hospital food.

Our reasons for establishing two different meals included the following. Because the test meal could be easily retrieved by the meal-recording app and the energy content was recorded for the food beforehand, we believed that the results would be almost the same if there were no problems with the app technique itself. Another reason that we used hospital food was that hospital food comes from a diet with minimal fat and salt, and we expected it to be prone to errors caused by the app. In fact, we compared the CVs for nutrients in the two test diets and found that they were generally approximately 0.1; however, the CV for the hospital diet was greater than 0.2, and as large as 0.5 for fats. Carbohydrates, such as rice, are also relatively simple and easy to evaluate quantitatively. The servings of proteins, such as meat and eggs, are also easy to visually evaluate, but the amount of oil in meat and the amount of salt used for seasoning are difficult to visually evaluate. Therefore, it is likely that there will be individual differences in the evaluation of the fat and salt contents of some dishes. Thus, these results suggested that it is difficult to evaluate each nutrient, and especially fat, in both apps. Moreover, the combined use of biomarkers, such as plasma glucose, urinary urea, urinary 3-methyl histidine, and urinary sodium levels, could also be useful for assessing the carbohydrate, protein, and salt contents [11,12,13]. For the fat contents, measuring the increase in the postprandial plasma triglyceride levels (chylomicron fraction) might be useful for estimating accurate fat intake that cannot be measured by apps [11]. Presumably, the combination with biomarkers will be important in correcting errors in the app measurement results.

The limitations of this study include a sex bias toward women and the small number of subjects. Interestingly, only 2 of the 7 men participated in the study, and 16 of the 18 women participated. One reason for the female bias is that women are more conscious about diet, weight, and other health issues than men. Because this study was unprecedented, it was not possible to statistically calculate the required number of participants, but even a small sample showed sufficiently significant differences that indicated that this number of participants was appropriate. Moreover, because this study was not designed to compare the two apps, no statistical comparisons were performed. In this study, the order of analysis was fixed as in the Asken and Calomeal apps, and so the performance of the two could not be compared as in the crossover test. The CV data suggest that there was no significant clinical difference between the two apps. Furthermore, because meal-recording apps combine multiple functions, it cannot be ruled out that different individuals may use different functions, which may affect the results. Including this possibility, it is a very interesting point that the total energy content of the hospital food was overestimated.

In conclusion, both apps were very accurate for the test meal, and the CVs for the total energy and each nutrient were generally 0.1. However, the CV for fat was particularly high for the hospital meal because the diet was designed to reduce fat and salt. Caution should be exercised when using a food-recording app to evaluate hospital diets that have been devised to limit salt and fat.

## Figures and Tables

**Table 1:** Linear-mixed-model results.

	Asken				Calomeal			
Energy								
	Estimate	2.50%	97.50%	Pr(>|t|)	Estimate	2.50%	97.50%	Pr(>|t|)
Meal	−0.249	−0.382	−0.115	0	−0.431	−0.599	−0.264	0
Time	0.113	0.052	0.174	0	0.079	0.005	0.152	0.039
Age (<or ≧45years)	−0.041	−0.106	0.025	0.222	−0.024	−0.106	0.059	0.558
MEAL*Time	−0.064	−0.101	−0.026	0.001	−0.044	−0.089	0.002	0.064
Carbohydrate								
	Estimate	2.50%	97.50%	Pr(>|t|)	Estimate	2.50%	97.50%	Pr(>|t|)
Meal	−0.172	−0.306	−0.037	0.014	−0.303	−0.485	−0.121	0.001
Time	0.071	0.009	0.133	0.027	−0.029	−0.113	0.056	0.509
Age (<or ≧45 years)	−0.051	−0.114	0.013	0.126	−0.025	−0.109	0.060	0.567
MEAL*Time	−0.043	−0.082	−0.004	0.034	0.011	−0.042	0.064	0.687
Fat								
	Estimate	2.50%	97.50%	Pr(>|t|)	Estimate	2.50%	97.50%	Pr(>|t|)
Meal	−0.171	−1.133	0.792	0.731	−0.335	−1.109	0.44	0.402
Time	0.810	0.360	1.260	0.001	0.857	0.502	1.211	0
Age (<or ≧45 years)	−0.171	−0.632	0.295	0.463	−0.126	−0.512	0.246	0.528
MEAL*Time	−0.409	−0.677	−0.141	0.003	−0.451	−0.673	−0.229	0
Protein								
	Estimate	2.50%	97.50%	Pr(>|t|)	Estimate	2.50%	97.50%	Pr(>|t|)
Meal	−0.147	−0.335	0.040	0.128	−0.588	−0.874	−0.302	0
Time	0.094	0.01	0.178	0.031	0.037	−0.094	0.169	0.58
Age (<or ≧45 years)	−0.017	−0.113	0.080	0.718	−0.012	−0.153	0.129	0.865
MEAL*Time	−0.033	−0.086	0.020	0.222	−0.022	−0.103	0.059	0.600
NaCl								
	Estimate	2.50%	97.50%	Pr(>|t|)	Estimate	2.50%	97.50%	Pr(>|t|)
Meal	−0.31	−0.581	−0.038	0.028	−0.706	−0.986	−0.426	0
Time	0.232	0.106	0.358	0	−0.02	−0.149	0.109	0.765
Age (<or ≧45 years)	−0.049	−0.18	0.082	0.439	−0.041	−0.192	0.111	0.576
MEAL*Time	−0.116	−0.195	−0.037	0.005	0.005	−0.074	0.084	0.899

MEAL*TIME means the interaction of MEAL and TIME.

## Data Availability

The data that support the findings of this study are available from the corresponding author, K.I., upon reasonable request.
